# Tumor-Secreted Exosomal lncRNA POU3F3 Promotes Cisplatin Resistance in ESCC by Inducing Fibroblast Differentiation into CAFs

**DOI:** 10.1016/j.omto.2020.05.014

**Published:** 2020-06-01

**Authors:** Yusuo Tong, Lili Yang, Changhua Yu, Weiguo Zhu, Xilei Zhou, Yaozu Xiong, Wanwei Wang, Fuzhi Ji, Dongcheng He, Xiufeng Cao

**Affiliations:** 1 Department of Radiation Oncology, The Affiliated Huaian No. 1 People’s Hospital of Nanjing Medical University, Huai’an, Jiangsu, China; 2 Department of Oncology Surgery, Nanjing First Hospital, Nanjing Medical University, Nanjing, China; 3 Department of Thoracic Surgery, Taikang Xianlin Drum Tower Hospital, School of Medicine, Nanjing University, Nanjing, China

## Abstract

Cancer-associated fibroblasts (CAFs), an activated subpopulation of fibroblasts, occupy a central position in the tumor microenvironment and have been shown to promote chemoresistance in multiple cancer types by secreting inflammatory cytokines. Herein, we report that tumor-secreted exosomal long non-coding RNAs (lncRNAs) can regulate cisplatin resistance in esophageal squamous cell carcinoma (ESCC) through transformation of normal fibroblasts (NFs) to CAFs. Primary CAFs and matched NFs were isolated from tumor tissues and matched normal esophageal epithelial tissues of ESCC patients. Fluorescence microscopy and qRT-PCR were used to investigate the transportation of exosomal lncRNAs from ESCC cells to NFs. To identify the specific lncRNAs involved, 14 ESCC-related lncRNAs were measured in NFs after incubation with exosomes from ESCC cells. We demonstrated that *lncRNA POU3F3* can be transferred from ESCC cells to NFs via exosomes and that it mediated fibroblast activation. Activated fibroblasts further promoted proliferation and cisplatin resistance of ESCC cells through secreting interleukin 6 (IL-6). Moreover, our clinical data showed that high levels of plasma exosomal *lncRNA POU3F3* correlated significantly with lack of complete response and poor survival in ESCC patients. Therefore, these data demonstrate that *lncRNA POU3F3* is involved in cisplatin resistance in ESCC and that this effect is mediated through exosomal *lncRNA POU3F3*-induced transformation of NFs to CAFs.

## Graphical Abstract

**Figure:**
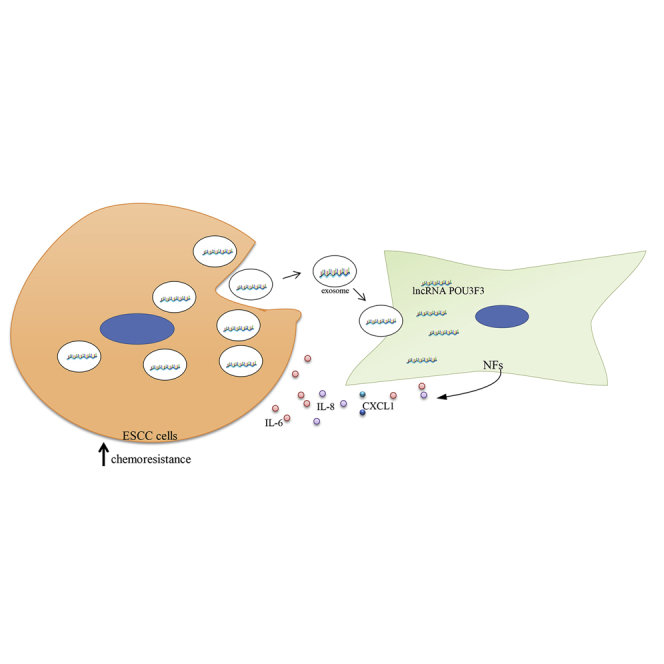

## Teaser

Cancer-associated fibroblasts are recruited and activated from nearby normal fibroblasts, and they create a microenvironment that promotes tumor chemoresistance. Cao and colleagues describe the role of tumor-secreted exosomal lncRNA in normal fibroblast activation in esophageal squamous cell carcinoma.

## Introduction

Esophageal squamous cell carcinoma (ESCC) is known as one of the most aggressive malignant tumors, with a 5-year survival rate of only 15%–25%.^1^ Patients with ESCC often present with a locally advanced stage, which is often refractory to conventional therapeutic approaches.^2^ For these patients, concurrent chemoradiotherapy (CCRT) has been widely used as the standard treatment.^3^ Some randomized clinical trials have shown that the addition of cisplatin-based chemotherapy with radiotherapy (RT) significantly improved 5-year survival rate compared with patients receiving RT alone.^4^ Despite the relatively prolonged overall survival times conferred by CCRT, the development of cisplatin resistance remains the main obstacles in the treatment of locally advanced diseases.^5^ Thus, it is urgent to clarify the molecular mechanism of cisplatin resistance and identify reliable biomarkers that can predict the CCRT response in ESCC patients.

Recently, the tumor microenvironment (TME) has gained increasing attention for its important roles in tumorigenesis, invasion, and drug resistance.^6^ Cancer-associated fibroblasts (CAFs), expressing α-smooth muscle actin (α-SMA), fibroblast activation protein (FAP), and fibroblast-specific protein 1, are the most abundant stromal cells in the TME.^7^, ^8^, ^9^ CAFs have been studied in multiple cancer types and found to have roles in creating extracellular matrix structure, activation of angiogenesis, and regulation of epithelial cell proliferation.^10^ In ESCC patients, the presence of FAP-positive CAFs in tumor stroma is correlated with lymph node metastasis and shorter disease-free survival.^11^ Several studies have demonstrated that CAFs are recruited and activated from nearby normal fibroblasts (NFs).^12^ Compared with NFs, CAFs significantly promote the proliferation, invasion, and chemoresistance in many cancer types.^13^^,^^14^ NFs exposed to tumor-conditioned medium (CM) assume a CAF-like phenotype; however, the exact mechanisms underlying activation of NFs by cancer cells remain largely unknown in ESCC.

Exosomes (30–150 nm) are microvesicles secreted by many cells types and can participate in cell-to-cell communication by transporting intracellular cargos.^15^ Secreted extracellular exosomes can carry multiple biologically active materials, including proteins, microRNAs (miRNAs), as well as long non-coding RNAs (lncRNAs) between cells.^16^ Tumor-secreted exosomal miRNAs are remarkably stable and could be a distant signal mediator. For instance, tumor-derived exosomal miR-1247-3p can convert fibroblasts to CAFs via downregulating B4GALT3.^17^ lncRNAs are defined as RNA transcripts longer than 200 nt with lack of protein-coding potential.^18^ Increasing evidence suggests that lncRNAs can modulate numerous hallmarks of cancer, including proliferation, metastasis, and chemoresistance.^19^, ^20^, ^21^ Tumor-secreted exosomes may be ideal lncRNA carriers and also provide a mechanism for transport of lncRNAs to the TME.^22^ However, whether tumor-secreted exosomal lncRNAs are involved in NF differentiation into CAFs have not been elucidated clearly in ESCC.

In this study, we aimed to identify the involvement of ESCC-secreted exosomal lncRNAs in fibroblast activation and to analyze their function in tumor progression and cisplatin resistance.

## Results

### Tumor-Secreted Exosomes Regulate Fibroblast Activation

**Figure 1:**
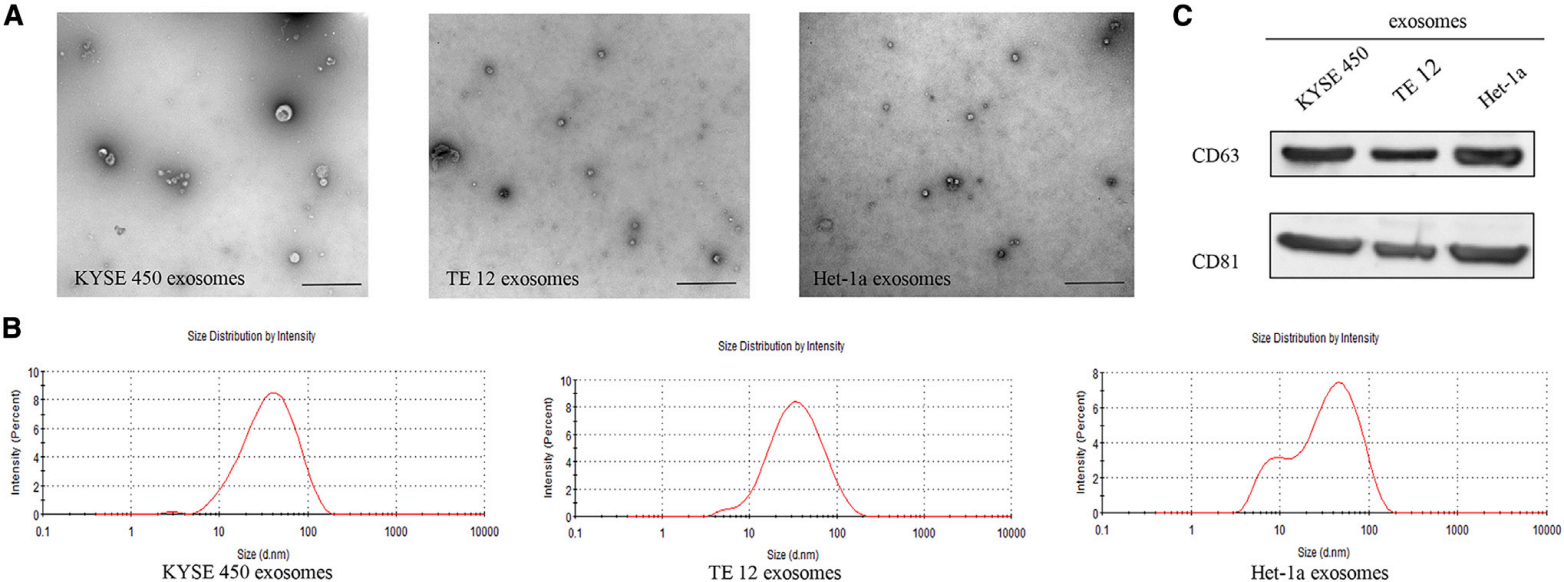
ESCC-Secreted Exosome Characterization (A) Representative electron microscopy images of the exosomes secreted by KYSE450, TE12, and Het-1a cells. Scale bars, 1 μm. (B) Size distribution of the isolated exosomes. (C) Western blot analysis of CD63 and CD81 in exosomes from different cells.

To determine whether ESCC cell-secreted exosomes participate in the activation of NFs, we chose two ESCC cell lines (KYSE450 and TE12) and normal esophageal epithelial cells (Het-1a) as models for studying tumor-secreted exosomes. As shown in Figures 1A and 1B, exosomes isolated from culture medium had a typical cup-shaped morphology, and their size was within the characteristic diameter range of 20–120 nm. Identity of purified exosomes was further confirmed by exosome-specific markers CD63 and CD81 (Figure 1C).

**Figure 2:**
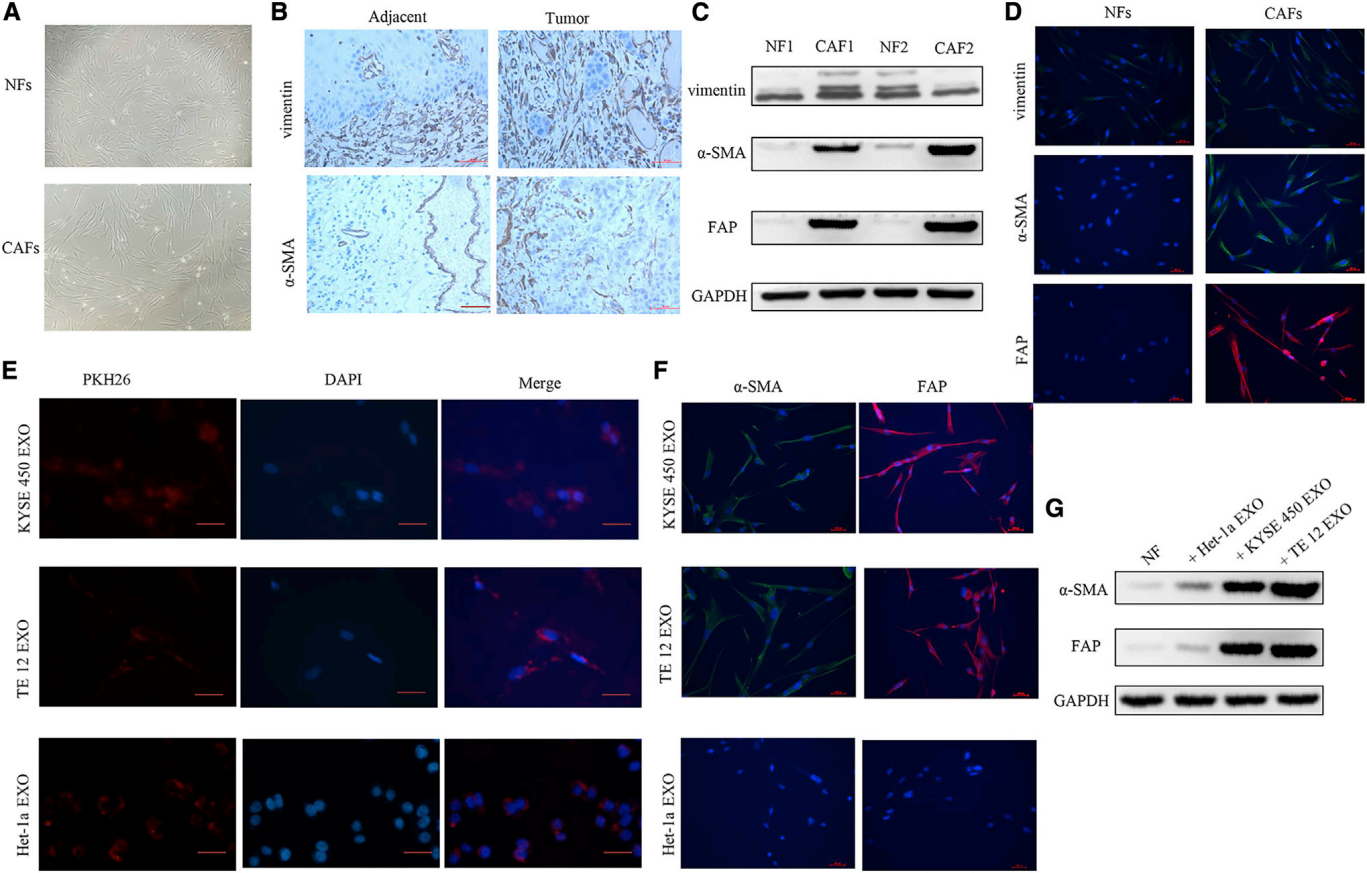
Exosomes Secreted from ESCC cells Induce NF Activation into CAFs (A) Representative morphology of NFs and CAFs isolated from patients with ESCC. (B) Representative immunohistochemical images of the NF marker (vimentin) and CAF marker (α-SMA) expression in ESCC tissues and adjacent normal tissues. Scale bars, 50 μm. (C) Western blot analysis of the expression of vimentin, α-SMA, and FAP in NFs and CAFs isolated from two pairs of esophageal cancer tissues and normal esophageal tissues. (D) Immunofluorescence staining of vimentin, α-SMA, and FAP in NFs and CAFs. Scale bars, 50 μm. (E) NFs were incubated with PKH26-labeled exosomes from KYSE450, TE12, or Het-1a cells for 24 h, and the red exosome signal was detected by fluorescence microscopy. Scale bars, 50 μm. (F) NFs were treated with KYSE450, TE12, or Het-1a cell-secreted exosomes (25 μg/mL) for 48 h. Fluorescence microscopy was used to detect the expression of α-SMA (green) and FAP (red) in NFs. Scale bars, 50 μm. (G) Western blot analysis of α-SMA and FAP expression in NFs treated with exosomes secreted from ESCC cells (25 μg/mL).

We focused on fibroblasts in this study for their crucial role in tumor progression and chemoresistance. Primary CAFs and NFs were isolated from fresh esophageal cancer tissues as well as matched normal tissues. As shown in Figure 2A, both NFs and CAFs showed bipolar and/or multipolar morphology. The immunohistochemistry (IHC) staining of vimentin and α-SMA revealed that NFs and CAFs were abundant in tumor stroma and adjacent normal tissues (Figure 2B). To further demonstrate that the cells we isolated were NFs and CAFs, fibroblast-specific biomarkers were examined by western blot analysis and immunofluorescence staining. As shown in Figures 2C and 2D, isolated NFs and CAFs both expressed vimentin, while only CAFs highly expressed α-SMA and FAP. To examine whether tumor-secreted exosomes could be transferred to NFs and promoted activation of NFs into CAFs, exosomes (25 μg/mL) were labeled with PKH26 and incubated with NFs for 24 h. As shown in Figure 2E, red fluorescence was observed in most NFs as indicated by fluorescence microscopy, without a significant difference between ESCC cell- and Het-1a cell-secreted exosomes. Expression of α-SMA and FAP is a defining characteristic of CAFs. We therefore measured the expression of α-SMA and FAP in exosome-educated NFs. We found that tumor-secreted exosomes strongly induced α-SMA and FAP expression compared with Het-1a cell-secreted exosomes (Figure 2F). These biomarker expression changes were further verified by western blot analysis. Incubation with exosomes from ESCC cells, but not those from Het-1a cells, significantly increased the expression of α-SMA and FAP in NFs (Figure 2G). Taken together, these results suggest that tumor-secreted exosomes promote fibroblast activation.

### *lncRNA POU3F3* in Exosomes Facilitates the Differentiation of NFs to CAFs

**Figure 3:**
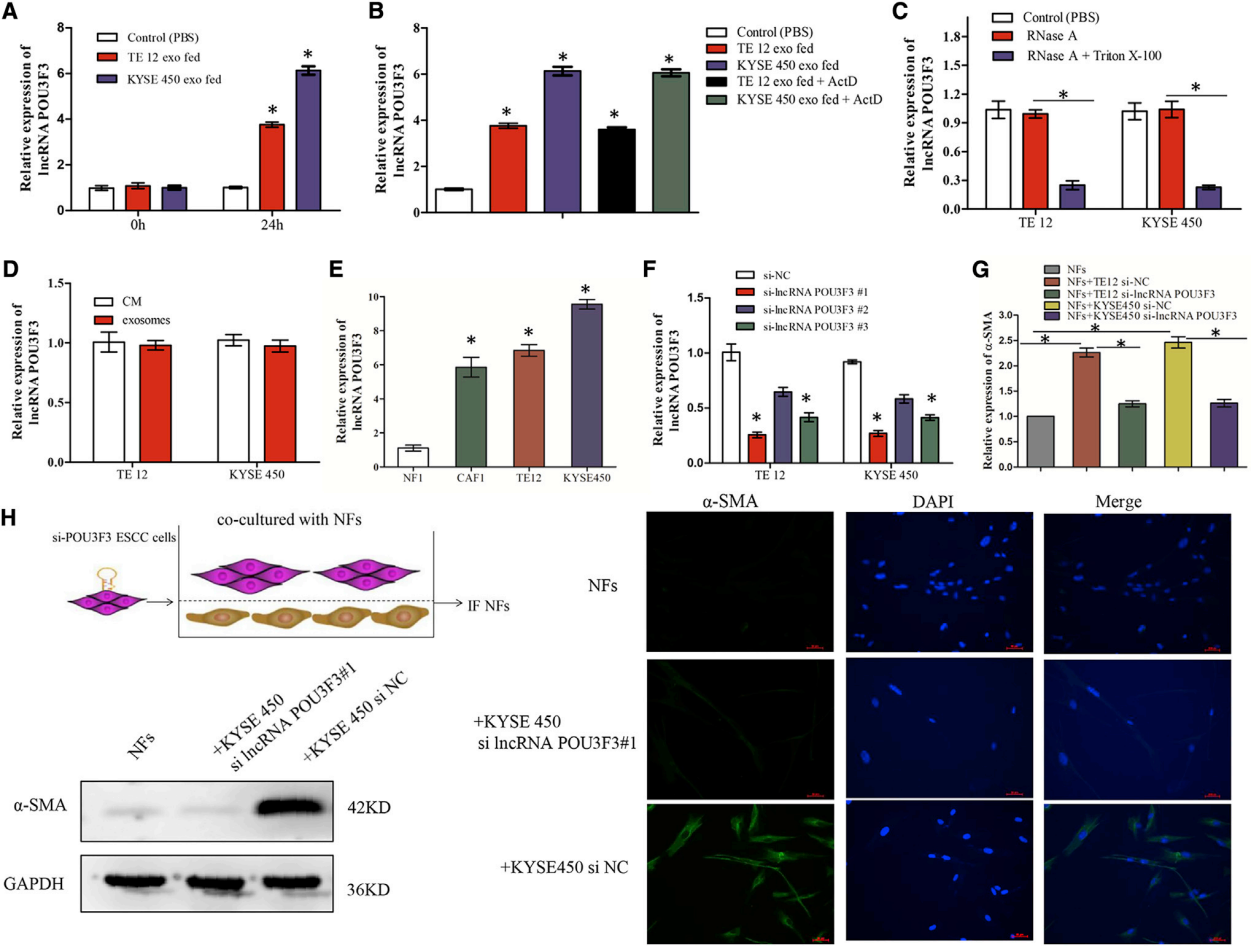
Tumor-Secreted Exosomal *lncRNA POU3F3* Facilitates the Differentiation of NFs into CAFs (A) qRT-PCR analysis of *lncRNA POU3F3* levels in NFs after incubation with KYSE450 or TE12 cell-secreted exosomes (or PBS as control) for 24 h. (B) qRT-PCR analysis of *lncRNA POU3F3* levels in NFs treated with actinomycin D (ActD, 1 μg/mL) followed by indicated exosome treatments for 24 h. (C) qRT-PCR analysis of *lncRNA POU3F3* expression in CM/cancer treated with RNase A alone (2 mg/mL) or combined with 0.1% Triton X-100 for 20 min. (D) qRT-PCR analysis of *lncRNA POU3F3* expression in exosomes and CM/cancer. (E) qRT-PCR analysis of *lncRNA POU3F3* expression in NF1, CAF1, and ESCC cells. (F) Knockdown efficiency of *lncRNA POU3F3*-specific siRNAs in KYSE450 and TE12. (G) qRT-PCR analysis of α-SMA expression in NFs co-cultured with ESCC cells (si-NC or si-lncRNA POU3F3), compared with monoculture. (H) Western blot analysis (left) and immunofluorescence (right) of α-SMA expression in NFs co-cultured with KYSE450 cells (si-NC or si-lncRNA POU3F3#1). Scale bars, 50 μm. Results are presented as mean ± SD. ∗p < 0.05.

Recent studies have suggested that exosomes can transfer lncRNAs from tumor cells to the non-malignant cells to modify the TME, and we therefore hypothesized that exosomal lncRNAs could facilitate the differentiation of NFs to CAFs. To identify the specific lncRNAs involved, we selected 14 ESCC-related lncRNAs (*AFAP1-AS1*, *CASC9*, *CCAT1*, *DNM3OS*, *FMR1-AS1*, *HNF1A-AS1*, *LINC01419*, *NMR*, *PART1*, *PCAT1*, *POU3F3*, *ROR*, *TTN-AS1*, and *TUG1*) for this study (Table 1). These lncRNAs were chosen based on their known associations with chemoresistance, survival, or progression in ESCC.^1^^,^^2^^,^^19^, ^20^, ^21^^,^^23^, ^24^, ^25^, ^26^, ^27^, ^28^, ^29^, ^30^, ^31^, ^32^, ^33^ We first incubated NFs with tumor-secreted exosomes (25 μg/mL) for 24 h. Using qRT-PCR, we found that the expression of *lncRNA POU3F3*, but not any other lncRNAs, was significantly increased in NFs after incubation with exosomes from KYSE450 or TE12 cells (Figure 3A; Table S1). In addition, the increase of *lncRNA POU3F3* level in NFs was not significantly affected by an RNA polymerase II inhibitor, excluding the involvement of endogenous induction (Figure 3B). We then examined the existing pattern of extracellular *lncRNA POU3F3*. The expression levels of *lncRNA POU3F3* in CM from ESCC cells (CM/cancer) were largely unchanged upon RNase A digestion but significantly declined when treated with RNase A and Triton X-100 simultaneously, suggesting that it was mainly encased within the membrane instead of directly secreted (Figure 3C). qRT-PCR analysis further confirmed that the *lncRNA POU3F3* level in exosomes was almost equal to that in CM/cancer, suggesting that exosomes were the main carrier for extracellular *lncRNA POU3F3* (Figure 3D). Next, we measured the expression of *lncRNA POU3F3* in ESCC cells, CAFs, and matched NFs. As expected, the expression of *lncRNA POU3F3* was much lower in NFs than in CAFs and ESCC cells (Figure 3E; Figure S1). Therefore, we selected *lncRNA POU3F3* for further study.

**Table 1:** Supporting Evidence for Selected lncRNAs

lncRNA	Association	References
*CASC9*	increased expression associated with poor survival and metastasis in ESCC	^1^^,^^24^
*PART1*	increased expression in serum exosomes associated with gefitinib resistance in ESCC	^2^
*CCAT1*	increased expression associated with TNM stage and poor survival in ESCC	^19^
*TTN-AS1*	promotes ESCC cell proliferation and metastasis	^20^
*TUG1*	promotes cisplatin resistance in ESCC cells	^21^
*AFAP1-AS1*	increased expression associated with cisplatin resistance and poor survival in ESCC	^23^
*DNM3OS*	increased expression associated with tumor progression in ESCC	^25^
*FMR1-AS1*	could be packaged into exosomes and released into the TME of ESCC	^26^
*HNF1A-AS1*	promotes cancer cells proliferation and migration in esophageal cancer	^27^
*LINC01419*	diminish the sensitivity of ESCC to 5-FU	^28^
*NMR*	inhibit cisplatin-induced apoptosis and increase drug resistance in ESCC	^29^
*PCAT1*	promotes ESCC cells proliferation through exosomes	^30^
*POU3F3*	promotes cell viability and proliferation in ESCC cells; could be released into serum of ESCC	^31^^,^^32^
*ROR*	promotes cancer cell proliferation and cisplatin resistance *in vitro*	^33^

TNM, tumor, node, metastasis; 5-FU, 5-fluorouracil.

To determine whether transferred *lncRNA POU3F3* could mediate fibroblast activation, we co-cultured NFs with *lncRNA POU3F3*-knockdown ESCC cells. Three different small interfering RNAs (siRNAs) were tested for knockdown efficiency, and the most effective si-lncRNA, POU3F3 #1, was selected for the following studies (Figure 3F; Figure S2). We found that NFs were activated after co-culture with si-NC ESCC cells, as seen by upregulation of α-SMA at both the mRNA and protein levels (Figures 3G and 3H). In contrast, we did not detect a significant increase in α-SMA expression in NFs after co-culture with *lncRNA POU3F3*-knockdown ESCC cells (Figures 3G and 3H). In addition, similar results were also observed when NFs were co-cultured with KYSE450 cells transfected with si-lncRNA POU3F3 #3 (Figure S3). Taken together, these results indicate that tumor-secreted exosomal *lncRNA POU3F3* facilitates the differentiation of NFs to CAFs.

### Activated Fibroblasts Promote Cisplatin Resistance in ESCC Cells

**Figure 4:**
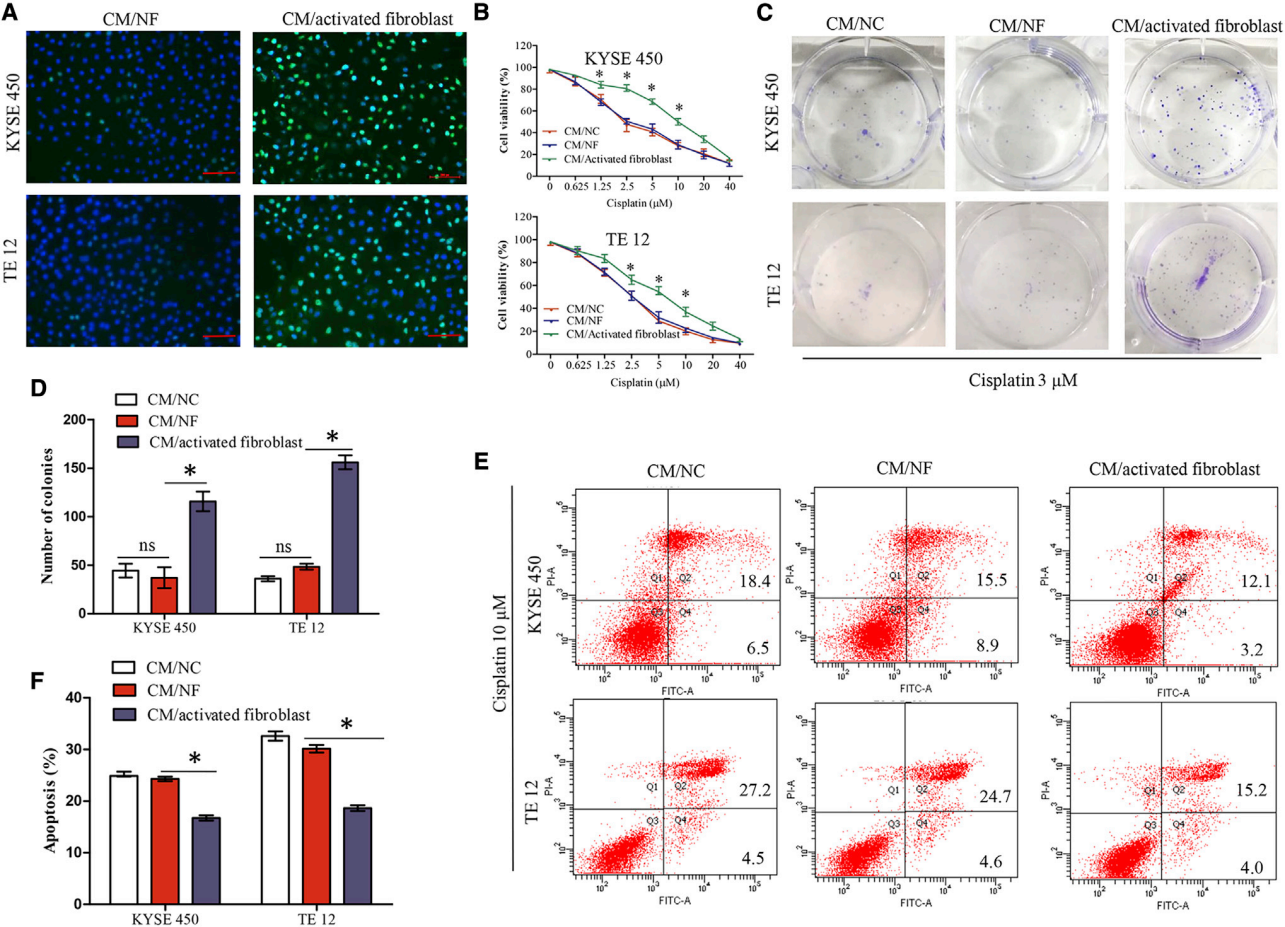
Fibroblasts Activated by *lncRNA POU3F3* Enhance Proliferation and Cisplatin Resistance of ESCC Cells *In Vitro* (A) KYSE450 and TE12 cells were grown in CM/NF or CM/activated fibroblast for 48 h, and cell proliferation was quantified using an EdU labeling assay. Representative micrographs are present. Scale bars, 100 μm. (B) KYSE450 and TE12 cells were cultured in CM/NC, CM/NF, or CM/activated fibroblast for 48 h. Cells were then treated with different concentrations of cisplatin for 6 h. Cell survival was determined by an MTT assay. (C and D) Plate colony formation assays of KYSE450 and TE12 cells cultured under the indicated CM containing cisplatin (3 μM). Culturing in CM/activated fibroblast promoted cisplatin resistance in KYSE 450 and TE 12. (E and F) KYSE450 and TE12 cells were cultured under indicated CM for 48 h. Cells were then treated with 10 μM cisplatin for 4 h, and cell apoptosis was analyzed by flow cytometry. Early and late apoptotic cells are shown in the right quadrant. Culturing in CM/activated fibroblast decreased cisplatin induced cell apoptosis in KYSE 450 and TE12. Results are present as mean ± SD. ∗p < 0.05, compared with the CM/NF. ns, not significant.

To evaluate the effect of activated fibroblasts on the proliferation of ESCC cells, we treated KYSE450 and TE12 cells with CM from activated fibroblasts (CM/activated fibroblast) or CM from NFs (CM/NF) for 48 h. The 5-ethynyl-2′-deoxyuridine (EdU) labeling assay showed that the ratio of EdU-positive cells in ESCC cells treated with CM/activated fibroblast was significantly enhanced when compared with CM/NF treatment (Figure 4A). Since NFs activated by *lncRNA POU3F3* significantly promoted ESCC cells proliferation, we speculated that activated fibroblasts might contribute to the chemoresistance of ESCC cells. Therefore, KYSE450 and TE12 cells were cultured in CM from normal controls (CM/NC), CM/NF, or CM/activated fibroblast for 48 h, and then sensitivity to cisplatin was determined by an MTT (3-(4,5-dimethylthiazol-2-yl)-2,5-dimethyltetrazolium bromide) assay. As shown in Figure 4B, CM/activated fibroblast significantly increased the half maximal inhibitory concentration (IC_50_) values in KYSE450 cells (13.57 versus 3.41 μM, p < 0.05) and TE12 cells (8.12 versus 2.94 μM, p < 0.05) as compared with CM/NF. Simultaneously, colony formation assays showed that compared with CM/NF, CM/activated fibroblast significantly promoted cisplatin resistance in KYSE450 and TE12 cells (Figures 4C and 4D). We next examined whether activated fibroblasts influenced cisplatin-induced cell apoptosis of ESCC cells. The flow cytometric analysis indicated that CM/activated fibroblast treatment significantly decreased cisplatin-induced tumor cell apoptosis as compared with the CM/NF (Figures 4E and 4F).

### Activated Fibroblasts Promote ESCC Cell Invasion and Migration *In Vitro*

**Figure 5:**
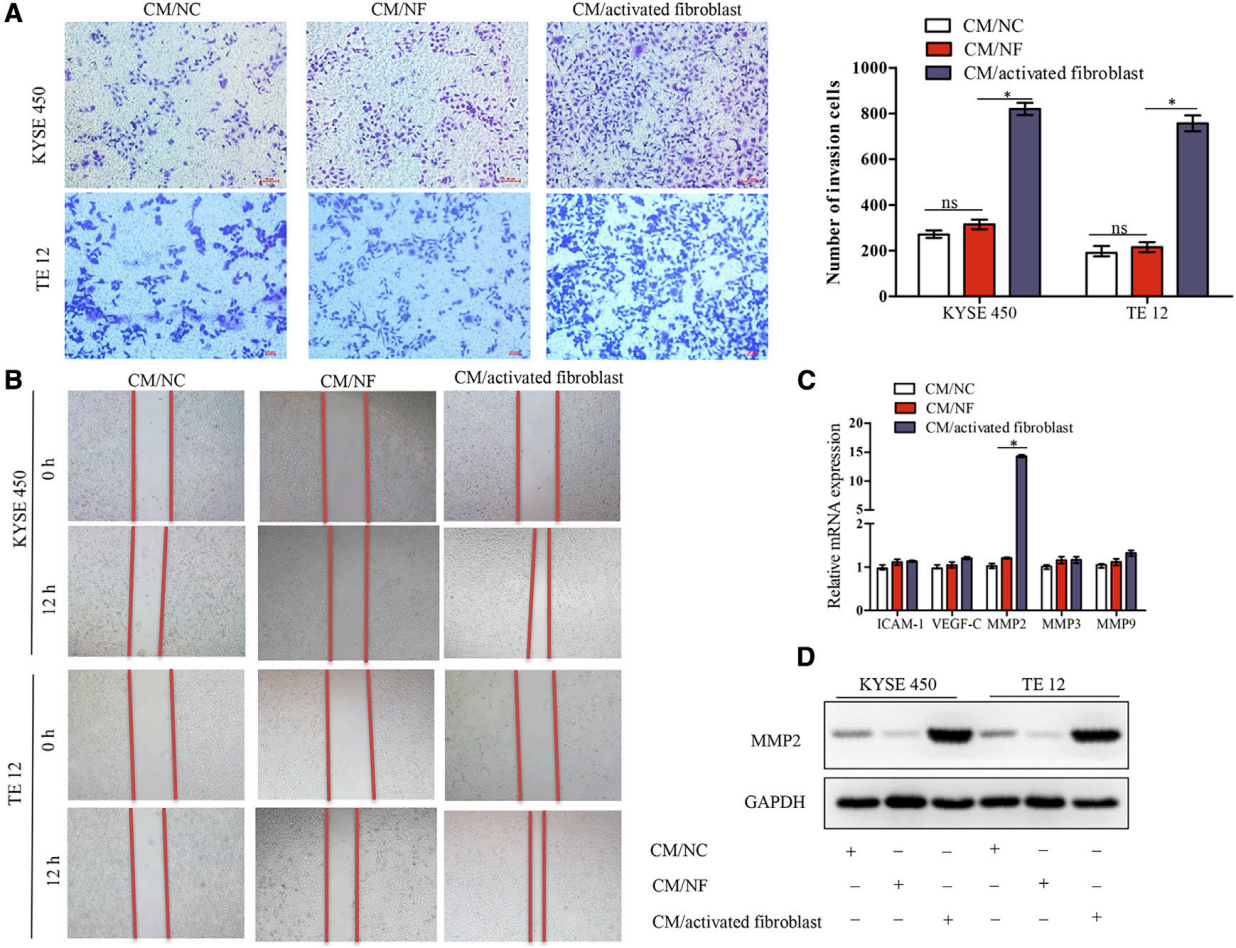
Fibroblasts Activated by *lncRNA POU3F3* Enhances the Migration and Invasion Abilities of ESCC Cells (A) Transwell assay of KYSE450 and TE12 cells treated with the indicated CM. Scale bars, 50 um. Results are present as mean ± SD. (B) Wound healing assay of KYSE450 and TE12 cells treated with the indicated CM. (C) qRT-PCR analysis of ICAM-1, VEGF-C, MMP2, MMP3, and MMP9 expression in KY450 and TE12 cells treated with CM/activated fibroblast. (D) Western blot analysis shows MMP2 overexpression in KY450 and TE12 cells treated with CM/activated fibroblast. ∗p < 0.05. ns, not significant

Besides cisplatin resistance, we explored whether activated fibroblasts influenced other malignant phenotypes such as the invasive and migration potential of ESCC cells. As shown in Figure 5A, after incubation in CM/activated fibroblast, the invasive ability of KYSE450 and TE12 cells in Matrigel-coated transwell chambers was significantly increased. Using a wound healing assay, we also found that two ESCC cell lines exhibited a faster wound closure rate in CM/activated fibroblast-treated cells than in CM/NF-treated cells (Figure 5B). To further determine the mechanism of activated fibroblasts on tumor invasion, we performed qRT-PCR to detect the expression levels of five genes (ICAM-1, VEGF-C, MMP2, MMP3, and MMP9) that were reported to be involved in ESCC invasion. As shown in Figure 5C, only MMP2 was found to be significantly altered among them. In addition, using western blot analysis, the MMP2 protein level was also increased in response to CM/activated fibroblast treatment (Figure 5D).

### Interleukin 6 (IL-6) Secreted by Activated Fibroblasts Promotes Cisplatin Resistance of ESCC Cells

**Figure 6:**
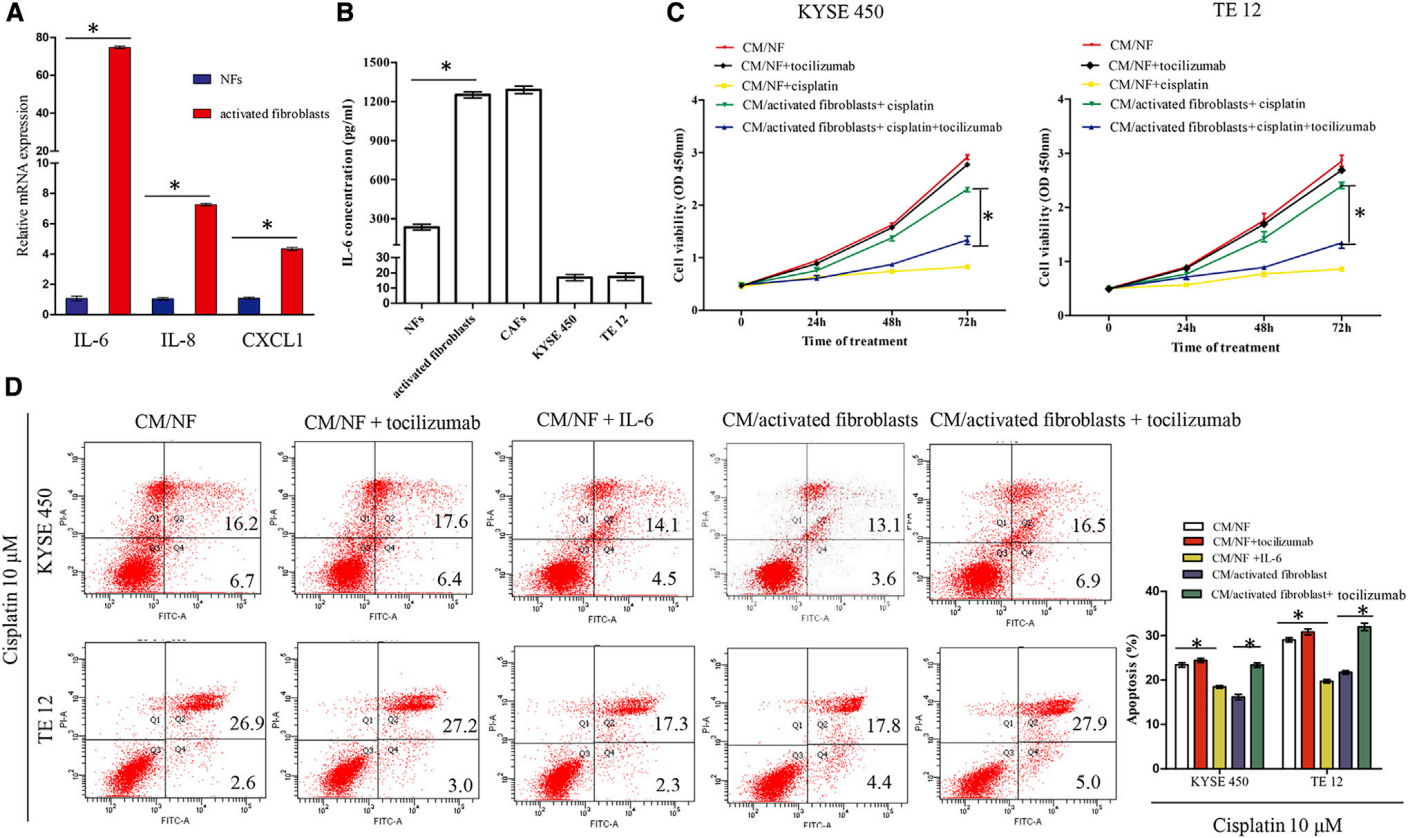
Activated Fibroblasts Regulate Chemoresistance through Secreting IL-6 (A) mRNA expression of *IL-6*, *IL-8*, and *CXCL1* in activated fibroblasts and NFs. (B) Expression level of IL-6 in the CM of NFs, activated fibroblasts, CAFs, and tumor cells, measured by ELISA. (C) ESCC cells were cultured in the indicated CM with or without IL-6 receptor-neutralizing antibody (tocilizumab) followed by treatment with 10 μM cisplatin. Cell viability was examined at the indicated time point using an MTT assay. (D) ESCC cells were cultured in CM/NF (control), CM/NF + tocilizumab, CM/NF + recombinant IL-6 (40 ng/mL), CM/activated fibroblast, or CM/activated fibroblast + tocilizumab followed by treatment with 10 μM cisplatin for 4 h, and cell apoptosis was analyzed by flow cytometric analysis. Early and late apoptotic cells are shown in the right quadrant. ∗p < 0.05.

The effects of CAFs on drug resistance have been shown to be mediated via paracrine secretion of many inflammatory cytokines. In this study, the CM collected from activated fibroblasts promoted cisplatin resistance of ESCC cells, suggesting that some of the inflammatory cytokine may play important roles in the cross-talk between activated fibroblasts and ESCC cells. In order to examine factors involved in cisplatin resistance of ESCC, we measured the expression levels of 7 genes (*IL-1β*, *IL-6*, *IL-8*, *IL-11*, *IL-32*, *HGF*, and *CXCL1*) that were reported to be secreted from CAFs in activated fibroblasts and NFs. By qRT-PCR analysis, we found that the mRNA levels of *IL-6*, *IL-8*, and *CXCL1* were significantly increased in activated fibroblasts compared with those in matched NFs (Figure 6A; Figure S4). Among these genes, *IL-6* showed an increase of 75-fold in activated fibroblasts, suggesting that IL-6 may be the key inflammatory cytokine to promote cisplatin resistance. Then, we performed ELISA analysis to further confirm the secretion. Similarly to CAFs, the concentrations of IL-6 were significantly higher in the supernatant from activated fibroblasts than those from matched NFs (Figure 6B). However, the concentrations of IL-6 were extremely low in the supernatants of KYSE450 and TE12 cells (Figure 6B).

To further evaluate the role of activated fibroblast-secreted IL-6 in cisplatin resistance, ESCC cells were cultured in CM/activated fibroblast in the presence or absence of IL-6 receptor neutralizing antibody (tocilizumab). As shown in Figure 6C, when KYSE450 or TE12 cells were cultured in CM/activated fibroblast, which contained 10 μg/mL tocilizumab, the cell viability after treatment with 10 μM cisplatin was significantly decreased, suggesting that activated fibroblast-secreted IL-6 was responsible for cisplatin resistance. Similar results were observed by the addition of 40 ng/mL recombinant human IL-6 to CM/NF (Figure 6D). Moreover, flow cytometry analysis also demonstrated that blocking IL-6 with tocilizumab could partially reverse the activated fibroblast-induced cisplatin resistance (Figure 6D). Collectively, these findings reveal that IL-6 secreted from activated fibroblasts promotes cisplatin resistance of ESCC cells.

### *lncRNA POU3F3* Levels in Plasma Correlate with CCRT Response and Survival in ESCC Patients

**Figure 7:**
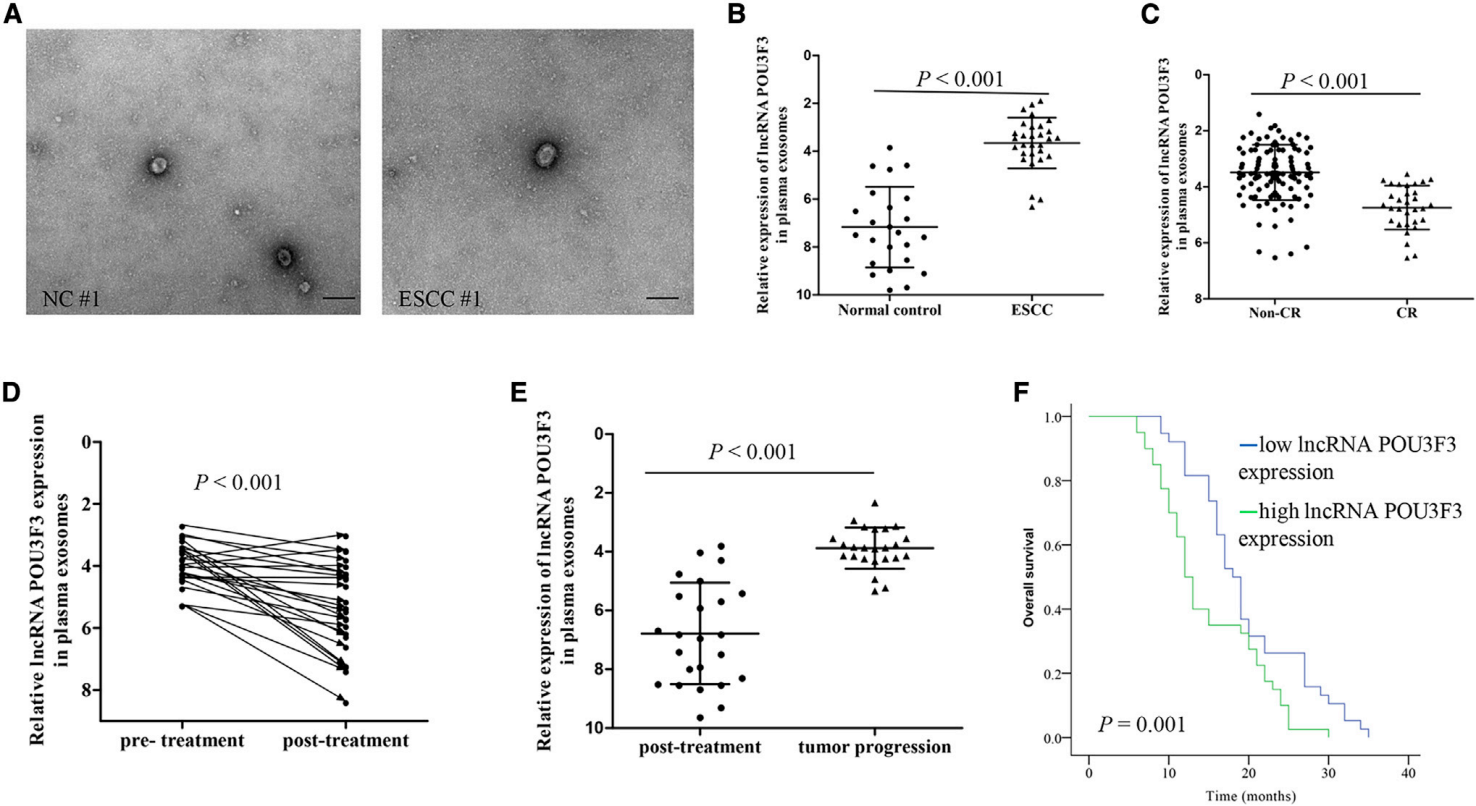
*lncRNA POU3F3* in Plasma Correlates with CCRT Response in ESCC Patients (A) Exosomes in normal control and ESCC plasma were detected by TEM. Scale bars, 200 nm. (B) *lncRNA POU3F3* expression levels in plasma exosomes from normal controls (n = 24) and ESCC patients (n = 30). (C) *lncRNA POU3F3* expression (ΔCt values) in pre-treatment plasma exosomes was significantly lower in patients with CR (n = 32) compared with patients with non-CR (n = 106). (D) ΔCt values of *lncRNA POU3F3* in matched plasma obtained from ESCC patients before and 4 weeks after CCRT (n = 24). (E) ΔCt values of *lncRNA POU3F3* in matched plasma obtained from ESCC patients 4 weeks after treatment and at the time of tumor progression (n = 27). (F) Kaplan-Meier analysis of overall survival based on plasma *lncRNA POU3F3* expression in ESCC patients (n = 78) with postoperative recurrence. The median *lncRNA POU3F3* expression was used as a cutoff. Survival data were analyzed by the Kaplan-Meier method and log rank test. In (B)–(D), results are presented as mean ± SD. A Student’s t test was used to analyze the data.

In light of these findings, we then determined whether cancer-secreted *lncRNA POU3F3* could serve as a predictive marker for response to cisplatin-based CCRT in ESCC. To explore this, we first observed plasma exosomes collected from NCs and ESCC patients by transmission electron microscopy (TEM). As shown in Figure 7A, most of the isolated exosomes exhibited typical cup-shaped morphology. Then, we measured the plasma exosomal *lncRNA POU3F3* expression in a small set of 24 healthy controls and 30 ESCC patients. In comparison with healthy controls, the expression levels of plasma exosomal *lncRNA POU3F3* were significantly increased in ESCC patients (p < 0.001, Figure 7B). We next investigated whether plasma exosomal *lncRNA POU3F3* could predict CCRT response in ESCC. A primary complete response (CR) was achieved in 25.3% (35/138) of the patients with ESCC. Our results showed that exosomal *lncRNA POU3F3* expression in pre-treatment plasma was significantly lower in patients who achieved a CR than in those without a CR (p < 0.001, Figure 7C). Furthermore, we also found that exosomal *lncRNA POU3F3* levels were decreased 4 weeks after CCRT, and they were upregulated again in patients with tumor progression (p < 0.001, Figures 7D and 7E). To further explore its clinical significance, we investigated exosomal *lncRNA POU3F3* expression in plasma samples obtained from 78 postoperative recurrent ESCC patients who received cisplatin-based combination chemotherapy. As shown in Figure 7F, patients with higher pre-treatment plasma exosomal *lncRNA POU3F3* expression had poorer overall survival (p < 0.001). Furthermore, our multivariable analysis showed that *lncRNA POU3F3* expression was the significant independent predictor of poor overall survival (Table S2). Collectively, our clinical data indicate that plasma exosomal *lncRNA POU3F3* can serve as a promising prognostic biomarker as well as an independent predictor of cisplatin resistance in ESCC.

## Discussion

Cisplatin resistance and the development of tumor recurrence are frequently observed in patients with locally advanced ESCC undergoing CCRT. Hence, a better understanding of the molecular mechanism of cisplatin resistance in ESCC is needed to improve prognosis. In this study, we first demonstrated that ESCC cells expressed significantly higher levels of *lncRNA POU3F3* than did NFs. We also found that *lncRNA POU3F3* could be transferred from cancer cells to NFs via exosomes and mediate NF activation. In addition, activated NFs can increase chemoresistance of ESCC cells to cisplatin by secreting IL-6. Furthermore, we identified that a higher expression of *lncRNA POU3F3* in plasma exosomes was associated with a poor response to cisplatin-based CCRT and short survival in patients with ESCC. Therefore, *lncRNA POU3F3* was verified to play an important role in the formation of cisplatin resistance.

Recent studies have described that exosomes released from cancer cells can be taken up by stromal cells and eventually modulate the biological functions in recipient cells. For example, Ringuette Goulet et al.^12^ found that exosomes released from bladder cancer cells can be transferred to fibroblasts and promoted the proliferation and expression of CAF markers. Another study indicated that melanoma cell-released exosomes can induce reprogramming of fibroblasts into CAFs and promote the expression of proangiogenic factors.^34^ These results suggested that tumor-secreted exosomes play an important role in fibroblast activation. In the TME, exosomes derived from tumor cells can communicate with fibroblasts by delivering oncogenic DNA, protein, and non-coding RNA. Several reports have described the role of exosomal miRNAs in transforming fibroblasts into CAFs.^34^^,^^35^ However, whether exosomal lncRNAs are involved in fibroblast activation remains poorly understood. In this study, we observed that ESCC cells could package *lncRNA POU3F3* into exosomes and actively secrete them in culture medium. By incubating NFs with exosomes from ESCC cells, we found that exosomal *lncRNA POU3F3* can be transferred to NFs. In addition, increased expression of *lncRNA POU3F3* in NFs could elevate α-SMA and FAP expression in recipient cells, suggesting that ESCC-secreted exosomal *lncRNA POU3F3* triggers NF reprogramming into CAFs.

Prior studies have shown that *lncRNA POU3F3* is expressed aberrantly and has oncogenic roles in several cancers. In our previous study, we observed that plasma *lncRNA POU3F3* levels were significantly elevated in ESCC patients compared with NCs and could be a potential marker for early detection of ESCC.^32^ In cancer types such as colorectal cancer and glioma, *lncRNA POU3F3* was overexpressed in tumor tissues and positively correlated with tumor grade. Knockdown of *lncRNA POU3F3* resulted in inhibition of cell proliferation.^36^^,^^37^ In ESCC, *lncRNA POU3F3* overexpression promoted cell proliferation by interacting with EZH2 to promote methylation of POU3F3.^31^ In contrast to the above reports implicating the direct enhancing effects of *lncRNA POU3F3* on cell survival, our data identified that *lncRNA POU3F3* can indirectly affect tumor growth by acting on the TME.

CAFs occupy a central position in the TME and have been reported to create suitable conditions for the progression of cancer cells.^38^^,^^39^ Furthermore, they are often the key factors that foster the resistance to therapy, whether it is chemotherapy, RT, or targeted approaches.^40^ For example, CAFs isolated from breast cancer tissues promote invasion and metastasis of breast cancer cells through integrin β3-p38 MAPK (mitogen-activated protein kinase) signaling.^41^ CAFs isolated from head and neck cancer are intrinsically resistant to cisplatin and can support cancer cell growth during cisplatin treatment.^42^ Our data demonstrated that NFs activated by *lncRNA POU3F3* enhanced the invasion and migration ability of ESCC cells by upregulation of MMP2. Generally, CAFs are recruited from resident fibroblasts, endothelial cells, mesenchymal stem cells, or bone marrow-derived progenitor cells.^43^^,^^44^ These cells are then activated by factors under the TME conditions, such as transforming growth factor β (TGF-β), platelet-derived growth factor, sonic hedgehog, and other genes to differentiate into CAFs.^44^, ^45^, ^46^ Using co-culture experiments, we observed in this study that NFs exhibited activated phenotypes, including the increased expression of α-SMA and FAP and the ability to promote ESCC cell proliferation. These increases were attenuated by silencing *lncRNA POU3F3* in ESCC cells using siRNA. As a result, we think that *lncRNA POU3F3* in the CM from ESCC cells mediated NF activation. Moreover, ESCC cells became insensitive to cisplatin after incubation with CM from activated fibroblasts.

Several recent studies have shown that CAFs confer resistance to anticancer drug therapy on tumor cells by the secretion of soluble factors, including IL-6, hepatocyte growth factor (HGF), TGF-β, IL-11, and others.^47^, ^48^, ^49^, ^50^ Among these, IL-6 plays a critical role in the communication between stromal cells and cancer cells in TME. IL-6 mediated cross-talk between CAFs and cancer cells not only by promoting tumor cell proliferation, but also by promoting fibroblast activation.^46^^,^^51^ It has also been reported to block apoptosis induced by p53 and certain anticancer drugs.^52^ In this study, we identified IL-6 as an important secreted factor from activated fibroblasts that promoted cisplatin resistance of ESCC cells. Blocking IL-6 with neutralizing antibody could partially reverse the activated fibroblast-induced cisplatin resistance. Significantly higher levels of IL-6 have been found in serum samples of patients with ESCC, breast cancer, and gastric cancer.^53^, ^54^, ^55^ In ESCC, higher levels of serum IL-6 were closely correlated with chemoresistance and poor survival.^56^^,^^57^ Recently, a growing number of studies showed that secreted IL-6 is involved in the development of chemoresistance via signaling pathways in the TME involving MAPK/signal transducer and activator of transcription 3 (STAT3), phosphatidylinositol 3-kinase (PI3K/AKT), or STAT3/ nuclear factor κB (NF-κB).^58^^,^^59^ IL-6 is produced by a variety of cells, including inflammatory cells, fibroblasts, endothelial cells, and cancer cells.^57^^,^^60^ In the TME, previous studies have been reported that IL-6 was mainly secreted from stromal cells.^61^ In bladder cancer, IL-6 correlated with CAF marker ACTA2 and negatively correlated with tumor purity, suggesting that CAFs were the main source of IL-6 in the TME.^62^ In this study, through the comparison of IL-6 protein expression between activated fibroblasts and ESCC cells, we found that IL-6 concentrations in activated fibroblasts were 80-fold greater than those in cancer cells. Thus, it is possible that increased expression of IL-6 by activated fibroblasts may contribute to cancer’s cisplatin resistance.

The results of the present study demonstrate that tumor-secreted exosomal *lncRNA POU3F3* can induce NF differentiation into CAFs. In addition, activated fibroblasts exhibit increasing secretion of IL-6, promoting cisplatin resistance of ESCC cells. More importantly, our findings demonstrate that *lncRNA POU3F3* could act as a clinical marker for the CCRT response in ESCC patients. These findings may improve the management of ESCC patients receiving cisplatin-based CCRT.

## Materials and Methods

### ESCC Patients and Clinical Samples

Blood samples were obtained from patients with locally advanced ESCC treated with CCRT between January 2017 and December 2018 at the Department of Radiation Oncology, Huai’an First Hospital, Nanjing Medical University. Patients were included when they had biopsy-confirmed ESCC and completed CCRT to 50.4 Gy (n = 138). Patients were divided into CR and less than CR according to the Response Evaluation Criteria in Solid Tumors (RECIST).^63^ The detailed clinical characteristics of these patients are summarized in Table S3. The study was approved by the Medical Ethics Committee of our institute, and written informed consent was obtained from all patients before CCRT.

For evaluation of the correlation between lncRNA expression and survival, plasma samples were collected from 78 ESCC patients with postoperative recurrence between January 2014 and December 2015. These patients received at least two cycles of cisplatin-based combination chemotherapy. Informed consent was obtained from each patient prior to participation in the study. The clinical information is provided in Table S4.

### Isolation and Culture of CAFs and Matched NFs

Primary CAFs and NFs were isolated from ESCC patients who had not been treated with preoperative CCRT before esophagectomy. In brief, CAFs were isolated from tumor tissues of ESCC, whereas NFs were isolated from matched non-tumor esophageal tissues at least 5 cm away from the tumor border. After washing with sterile PBS, tissue specimens were minced into 1- to 3-mm^3^ fragments and then digested with 1 mg/mL collagenase I (Sigma) at 37°C for 2 h with 5% CO_2_. After filtration and centrifugation (1,000 rpm for 5 min), the cell precipitation was seeded into T25 tissue culture flask. After a 30 min incubation, the medium was replaced with fresh medium to remove nonadherent cells (mainly tumor cells). Then, the fibroblasts were cultured in RPMI 1640 (Gibco) with 10% fetal bovine serum (FBS) (Gibco) and antibiotics (100 U/mL penicillin and 100 μg/mL streptomycin, Gibco). CAFs and NFs were further identified by the presence of fibroblast-specific markers (α-SMA, FAP, and vimentin). The fibroblasts for experiments were used between passages 3 and 10. Two pairs of CAFs and matched NFs (CAF-1 and CAF-2 and matched NF-1 and NF-2) were finally isolated and used in this study. Written informed consent was obtained from each patient prior to tumor sample collection.

### Cell Lines

The human ESCC cell lines (KYSE450 and TE12) and normal esophageal epithelial cells (Het-1a) were a generous gift from Dr. Zhi-Hua Liu (Chinese Academy of Medical Sciences, Beijing, China). All cell lines were maintained in RPMI 1640 with 10% exosome-free FBS (Gibco) at 37°C in a 5% CO_2_ atmosphere.

### Preparation of the CM

To obtain CM/cancer, CM/CAF, and CM/NF, cells were planted in 75-cm culture flasks with RPMI 1640 with 10% FBS. Supernatants were collected after 48 h (cells at 80% confluence) and centrifuged (3,000 rpm for 30 min at 4°C). NFs were incubated in CM/cancer for 48 h, and the resulting CM was collected and defined as CM/activated fibroblast. Fresh RPMI 1640 with 10% FBS was defined as CM/control.

### Immunofluorescence Staining

Cells were seeded in 24-well plates and incubated overnight. After the indicated treatment, cells were fixed with 4% paraformaldehyde, permeabilized with 0.2% Triton X-100, and blocked with 5% bovine serum albumin. The cells were then incubated with anti-human antibody against vimentin (Cell Signaling Technology, 5741S, 1:100), FAP (Abcam, ab28244, 1:100), and α-SMA (Abcam, ab5694, 1:200) overnight at 4°C, followed by incubation with Alexa Fluor 488- or Alexa Fluor 555-conjugated secondary antibody (Cell Signaling Technology) at room temperature for 30 min. Cells were observed and imaged using a fluorescence microscope (Nikon Eclipse Ti, Japan).

### Exosome Isolation and Characterization

Exosomes were extracted from plasma samples using an ExoQuick plasma preparation and exosome precipitation kit (System Biosciences) according to the manufacturer’s instructions. In addition, exosomes were isolated from CM by the differential centrifugation method. Briefly, after cell cultures reached 90% confluency, medium was harvested and centrifuged at 300 × *g* for 5 min, 3,000 × *g* for 30 min, and 10,000 × *g* for 30 min at 4°C to remove debris and apoptotic bodies. Subsequently, the exosomes were pelleted by centrifugation at 100,000 × *g* for 80 min at 4°C. The purified exosomes were resuspended in PBS for cell treatment or used for RNA/protein extraction. The protein concentration was measured by using a bicinchoninic acid (BCA) protein assay kit (Thermo Fisher Scientific). The size and quality of the exosomes were determined using a Zetasizer Nano ZS instrument (Malvern Instruments, UK).

### Exosome Uptake by Fibroblasts

Isolated exosomes were pre-labeled with a PKH26 fluorescent cell linker kit (Sigma-Aldrich) following the manufacturer’s procedures. Subsequently, NFs were seeded in a 24-well plate and incubated with labeled exosomes (25 μg/mL) for 24 h. The cells were then prepared for immunofluorescence as described above.

### MTT assay

An MTT assay (Sigma) was performed to calculate the IC_50_ for cisplatin. Briefly, ESCC cells after corresponding treatment were seeded in 96-well plates (3 × 10^3^ cells/well) and incubated with serially diluted cisplatin (0, 0.625, 1.25, 2.5, 5, 10, 20, and 40 μmol/L) for 6 h. Then, the medium was replaced with 100 μL of fresh medium. After incubation for 48 h, 10 μL of MTT was added and the plate was incubated at 37°C for 3 h. Absorbance at 490 nm was then measured by a multi-well plate reader (Tecan). The IC_50_ of cisplatin was estimated by the dose-response curve.

### EdU Labeling Assay

The effect of activated fibroblasts on KYSE450 and TE12 cell proliferation was examined using a Cell-Light EdU Apollo 488 *in vitro* imaging kit (Ribobio, China). Briefly, ESCC cells (5 × 10^3^ cells/well) were seeded in 96-well plates and cultured in CM/NF or CM/activated fibroblast for 48 h. Then, the tumor cells were incubated with EdU labeling medium. After a 2-h incubation, the cells were fixed with 4% paraformaldehyde and permeabilized with 0.5% Triton X-100. Then, the cells were stained with Apollo 488 for 30 min. Subsequently, the cells were counterstained with Hoechst 33342 and analyzed using a fluorescence microscope (Nikon Eclipse Ti).

### Cell Apoptosis Assay

The apoptosis assay was performed using an annexin V-fluorescein isothiocyanate (FITC) and propidium iodide (PI) apoptosis kit (KeyGen Biotech, China). In brief, ESCC cells were plated in six-well plates and incubated with CM/NC, CM/NF, or CM/activated fibroblast for 48 h. After 24 h of 10 μM cisplatin treatment, the cells were collected and stained with 5 μL of FITC and 5 μL of PI. After incubation for 15 min, each sample was then analyzed by flow cytometry (BD FACSCanto II).

### Transient Transfection and Co-culture Assay

*lncRNA POU3F3*-specific siRNAs were synthesized by Ribobio (Guangzhou, China). Lipofectamine 2000 (Invitrogen) was used for transfection following the manufacturer’s instructions. The target sequences for *lncRNA POU3F3* siRNAs were (#1, 5′-CAGTGTGAAGGGAAGGCTA-3′, #2, 5′-GGTGCTGGAGAGTTGA GAA-3′, #3, 5′-GCTGGAGAGTTGAGAA TAT-3′).

Co-culture experiments were performed using transwell membranes (0.4-μm pores, Corning Life Sciences). Approximately 1 × 10^5^ ESCC cells (si-NC or si-lncRNA POU3F3) were plated in the upper chamber, and 5 × 10^4^ NFs were seeded on the bottom of the 12-well plate. After incubation for 72 h, NFs were collected for RNA extraction or further cytological experiments.

### RNA Extraction and qRT-PCR

Total RNA was extracted from tumor tissues and culture cells using TRIzol reagent (Invitrogen). RNA from plasma or culture medium was extracted using a mirVana Paris kit (Ambion, USA). qRT-PCR was conducted using SYBR Premix Ex Taq II (Takara, China) according to the manufacturer’s instructions on the ABI 7500 real-time PCR system (Applied Biosystems, Foster City, CA, USA). Each sample was performed in triplicate. GAPDH was used to normalize mRNA expression levels. All of the primers used in this study are listed in Table S5.

### Western Blotting

Exosomes and cells were lysed using radioimmunoprecipitation assay (RIPA) buffer containing protease inhibitors (KeyGen Biotech) and centrifuged at 4°C for 5 min. Protein was separated on SDS-PAGE gel with various concentrations depending on the molecular weight of the protein and transferred onto polyvinylidene fluoride (PVDF) membranes (Merck Millipore). To detect the indicated proteins, primary antibodies were used as follows: vimentin (Cell Signaling Technology, 5741S, 1:1,000), FAP (Abcam, ab28244, 1:1,000), α-SMA (Abcam, ab5694, 1:500), CD63 (Abcam, ab134045, 1:1,000), CD81 (Abcam, ab79559, 1:1,000), and GAPDH (Cell Signaling Technology, #2118, 1:1,000).

### ELISA analysis

IL-6 levels in culture supernatants were quantified using ELISA kits (Beyotime) according to the manufacturer’s instructions. All samples were analyzed in triplicate.

The detailed methods for TEM, plate colony formation assays, wound healing assays, cell invasion assays, and IHC are described in Supplemental Materials and Methods.

### Statistical Analysis

Statistical analyses were performed using SPSS 20.0 software. The results are presented as mean ± SD. Data normality was assessed using Kolmogorov-Smirnov tests. A Student’s t test was used to assess differences between groups. Survival analysis was calculated using the Kaplan-Meier method. A p value less than 0.05 was considered statistically significant.

## Author Contributions

X.C., Y.T., and L.Y. conceived and designed the experiments. X.Z., Y.X., F.J., W.W., and D.H. were responsible for providing the clinical samples. Y.T. and L.Y. performed the experiments. W.Z. and C.Y. analyzed and interpreted the data.

## Conflicts of Interest

The authors declare no competing interests.

## Supplemental Information

Document S1. Figures S1–S4, Tables S1–S5, and Supplemental Materials and Methods

Document S2. Article plus Supplemental Information
