# Supplementary material for: Tumor-Secreted Exosomal lncRNA POU3F3 Promotes Cisplatin Resistance in ESCC by Inducing Fibroblast Differentiation into CAFs
Source: Mol Ther Oncolytics. 2020 Jun 1;18:1–13. doi: 10.1016/j.omto.2020.05.014 (PMC7321817; doi:10.1016/j.omto.2020.05.014)
Supplement: Document S1. Figures S1–S4, Tables S1–S5, and Supplemental Materials and Methods [file mmc1.pdf]

OMTO, Volume *18*

Supplemental Information

Tumor-Secreted Exosomal lncRNA POU3F3 Promotes Cisplatin Resistance in ESCC by Inducing Fibroblast Differentiation into CAFs

Yusuo Tong, Lili Yang, Changhua Yu, Weiguo Zhu, Xilei Zhou, Yaozu Xiong, Wanwei Wang, Fuzhi Ji, Dongcheng He, and Xiufeng Cao

Supplemental Information

Figure S1

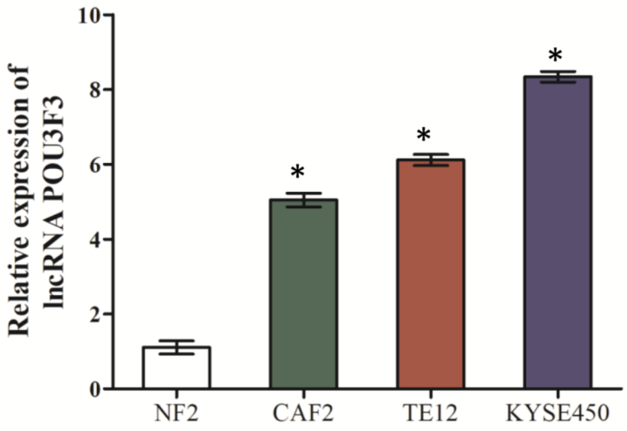

Figure S2

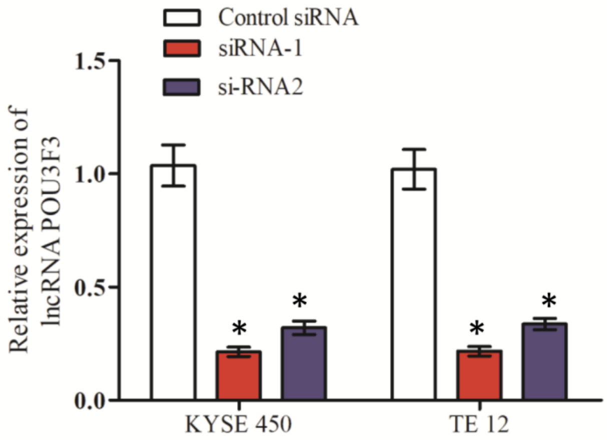

Figure S3

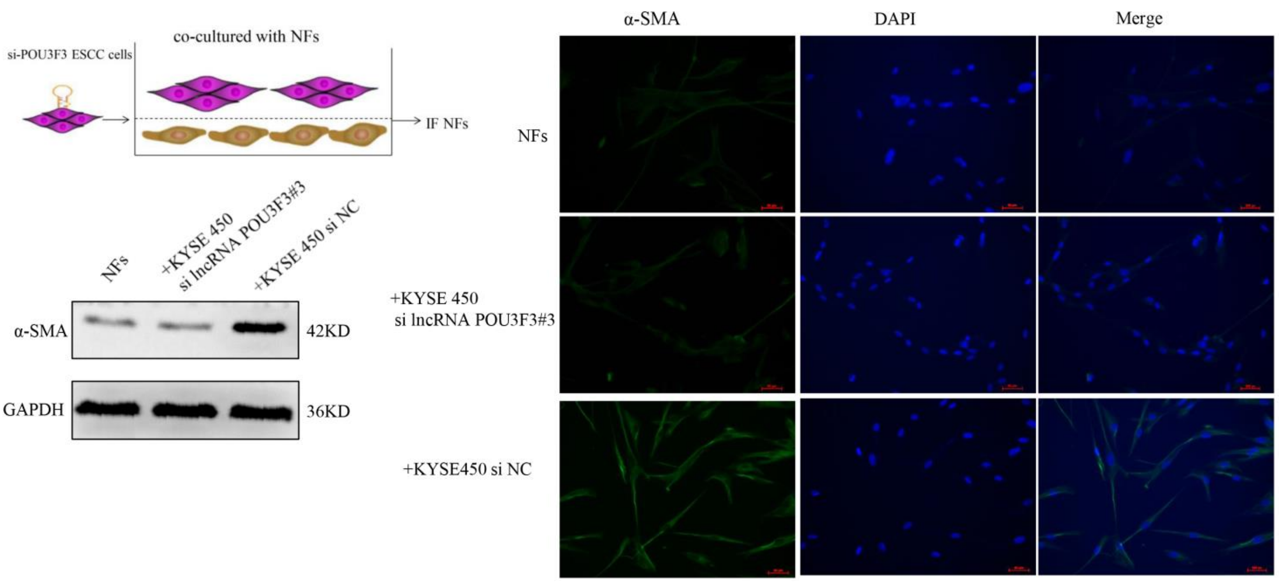

Figure S4

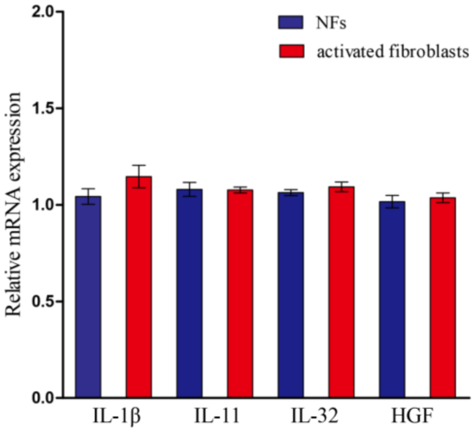

Figure S1 qRT-PCR analysis of *lncRNA POU3F3* expression in NF2, CAF2, and ESCC cells.

Figure S2 qRT-PCR analysis of *lncRNA POU3F3* in exosomes isolated from the CM of KYSE 450 and TE 12 cells after transfection with control or lncRNA POU3F3 siRNA.

Figure S3 Western blot analysis (left) and Immunofluorescence (right) of α-SMA expression in NFs co-cultured with KYSE 450 (si-NC or si-lncRNA POU3F3#3).

Figure S4The mRNA expression of *IL-1β*, *IL-11 IL-32*, and *HGF* in activated fibroblasts and NFs.

Table S1. ESCC-related lncRNAs expression in NFs after ESCC-secreted exosome treatment

lncRNA	Average fold change
AFAP1-AS1	1.05
CASC9	1.22
CCAT1	1.13
DNM3OS	1.17
FMR1-AS1	1.14
HNF1A-AS1	0.97
LINC01419	1.08
NMR	1.26
PART1	1.17
PCAT1	1.11
ROR	1.08
TTN-AS1	1.31
TUG1	1.24

Average fold was calculated from the delta-delta Ct value of 3 expts

Table S2 Univariate and multivariate analysis of OS of patients treated with cisplatin-based combination chemotherapy (78 cases)

Variables	Univariate analysis		Multivariate analysis	
	Median OS (months)	*p*-value	HR (95% CI)	*p*-value
Age (y)		0.316		
<60	13			
≥60	14			
Sex		0.703		
Female	15			

Male	12			
Tumor length (cm)		0.630		
≤ 5	15			
> 5	13			
Histological grade		0.304		
G1-G2	14			
G3-G4	14			
TNM stage (7th ed)		0.543		
II	15			
III	14			
Recurrence site		0.030	1.570 (0.902-2.734)	0.111
Locoregional recurrence	15			
Distant metastasis	9			
Chemotherapy		0.146		
Cisplatin + paclitaxel	12			
Cisplatin + docetaxel	14			
Chemotherapy cycles		0.929		
<4	12			
≥4	16			
lncRNA POU3F3 expression		0.001	2.206 (1.339-3.635)	0.002
Low	18			
High	12			

Table S3 Clinical characteristics of 138 ESCC patients treated with CCRT

Variables	*lncRNA POU3F3* expression		[a]*p* value
	Low (n=69)	High (n=69)	
Age (years)			0.404
≤ 60	17 (24.6%)	12 (17.4%)	
>60	52 (75.4%)	57 (82.6%)	
Sex			0.561
Female	16 (23.1%)	20 (28.9%)	
Male	53 (76.9%)	49 (71.1%)	
Tumor location			0.359
Upper third	10 (14.5%)	16 (23.2%)	
Middle third	48 (69.6%)	41 (59.4%)	
Lower third	11 (15.9%)	12 (17.4%)	
Tumor length (cm)			0.864
≤ 5	32 (46.3%)	30 (43.5%)	
> 5	37 (53.7%)	39 (56.5%)	
Histological grade			0.649
Well/moderate	59 (85.5%)	56 (81.2%)	
Poor	10 (14.5%)	13 (18.8%)	

T factor			0.214
T2	16 (23.2%)	12 (17.4%)	
T3	46 (66.7%)	43 (62.3%)	
T4	7 (10.1%)	14 (20.3%)	
N factor			0.348
Negative	23 (33.3%)	17 (24.6%)	
Positive	46 (66.7%)	52 (75.4%)	
Clinical stage (6th ed)			0.179
II	30 (43.5%)	22 (31.9%)	
III	36 (52.2%)	39 (56.5%)	
IVa	3 (4.3%)	8 (11.6%)	
Clinical response			0.018
CR	24 (34.8%)	11 (15.9%)	
Less than CR	45 (65.2%)	58 (84.1%)	

Abbreviations: CR: complete response

[a]*p* value was calculated by χ^2 test or Fisher's exact test

Table S4 Clinical information of 78 ESCC patients in the survival analysis

Variables	Patients (n = 78) No. (%)
Age (y)	
Median	57
Range	48-73
Sex	
Female	27 (34.6%)
Male	51 (65.4%)
Location of primary tumor	
Cervical	1 (1.3%)
Upper thoracic	9 (11.5%)
Middle thoracic	52 (66.7%)
Lower thoracic	16 (20.5%)
Tumor length (cm)	
≤ 5	29 (37.2%)
> 5	49 (62.8%)
Histological grade	
G1-G2	59 (75.6%)
G3-G4	19 (24.4%)
T factor	
T2	12 (15.4%)
T3	43 (55.1%)
T4	23 (29.5 %)

N factor	
N0	29 (37.3%)
N1	31 (39.7%)
N2	14 (17.9%)
N3	4 (5.1%)
TNM stage (7th ed)	
II	28 (35.9%)
III	50 (64.1%)
Recurrence site	
Locoregional recurrence	61 (78.2%)
Distant metastasis	17 (21.8%)
Chemotherapy	
Cisplatin + paclitaxel	13 (16.7%)
Cisplatin + docetaxel	65 (83.3%)
Chemotherapy cycles	
Median	4
Range	2-6

Table S5 Sequences of primers used for qRT-PCR in this study

Genes	Primer sequence (5’-3’)
AFAP1-AS1_F	TCGCTCAATGGAGTGACGGCA
AFAP1-AS1_R	CGGCTGAGACCGCTGAGAACTT
CASC9_F	TTGGTCAGCCACATTCATGGT
CASC9_F	AGTGCCAATGACTCTCCAGC
CCAT1_F	TCACTGACAACATCGACTTTGAAG
CCAT1_R	GGAGAAAACGCTTAGCCATACAG
DNM3OS_F	ATAGAGCAAGTCTGGATT
DNM3OS_R	GGATGAGGCAATAACATT
FMR1-AS1_F	ACCAAACCAAACCAAACCAA
FMR1-AS1_R	GTGGGAAATCAAATGCATCC
HNF1A-AS1_F	TCAAGAAATGGTGGCTAT
HNF1A-AS1_R	GCTCTGAGACTGGCTGAA
LINC01419_F	GAAACTCCGAACACATCTG
LINC01419_R	TTCTCCTGCTGGTTGATT
PART1_F	AAGGCCGTGTCAGAACTCAA
PART1_R	GTTTTCCATCTCAGCCTGGA
PCAT1_F	TGAGAAGAGAAATCTATTGGAACC
PCAT1_R	GGTTTGTCTCCGCTGCTTTA
POU3F3_F	AATCACTGCAATTGAAGGAAAAA
POU3F3_R	CCTTGTTTTCCAACCCTTAGACT
ROR_F	TTCAGTTCCCTAAAGTCACCC
ROR_R	GTCCTTCTAAGCCTCTGTTGC
TTN-AS1_F	TTAGCGCAGCTCTCCTTCAC

TTN-AS1_R	AAGCAACACCGCAGTTCCAT
TUG1_F	CAAGAAACAGCAACACCAGAAG
TUG1_R	TAAGGTCCCCATTCAAGTCAGT
IL-1β_F	ATGATGGCTTATTACAGTGGCAA
IL-1β_R	GTCGGAGATTCGTAGCTGGA
IL-6_F	ACTCACCTCTTCAGAACGAATTG
IL-6_R	CCATCTTTGGAAGGTTCAGGTTG
IL-8_F	TTTTGCCAAGGAGTGCTAAAGA
IL-8_R	AACCCTCTGCACCCAGTTTTC
IL-11_F	CGAGCGGACCTACTGTCCTA
IL-11_R	GCCCAGTCAAGTGTCAGGTG
IL-32_F	ACGACTTCAAAGAGGGCTAC
IL-32_R	TCCTCAACATCCGGGACAGG
HGF_F	CAAGGACCTACGAGAAAATTAC
HGF_R	ATCACAGTTTGGAATTTGGG
CXCL1_F	CTTGCCTCAATCCTGCATC
CXCL1_R	CCTTCTGGTCAGTTGGATTTG
GAPDH_F	TGCACCACCAACTGCTTAGC
GAPDH_R	GGCATGGACTGTGGTCATGAG

Supplemental Methods

Transmission electron microscope

For electron microscope analysis, exosomes were deposited onto electric microscope grids and then negatively stained with 2% phosphotungstic acids for 5 min. After air-dried for 15 min, samples were observed using a JEM-1010 transmission electron microscope (JEOL, Japan) at 80 kV accelerating voltage.

Colony formation assay

KYSE 450 and TE 12 cells (5×10^2) were seeded in 6-well plates in CM/NF or CM/activated fibroblast. Twenty-four hours later, 3 μM cisplatin was added. After 14 d, cells were stained with 0.1% crystal violet (Beyotime) and the number of colonies (> 50 cells/colony) was counted.

Wound healing assay

ESCC cells were plated in 6-well plates, pre-treated as indicated, and cultured to confluency. Then a 10 μl pipette tip was used to create a scratch on the cell monolayers. Wound sizes were verified with an ocular ruler to ensure that each sample has the same initial wound width. Photos were taken at 0, 12, and 24 h.

Cell invasion assay

For invasion assay, ESCC cells (4×10^5) were suspended in 200 μl FBS free medium and plated on the matrigel-coated membrane (Corning) in the upper chamber (8μm pore size, Corning). Medium in the lower chamber was CM/NC, CM/NF, or CM/activated fibroblast. After 24 h of incubation, cells on the low side of the membrane were fixed using methanol and stained with 0.1% crystal violet.

Immunohistochemistry (IHC)

IHC staining was performed to detect the expression of α-SMA and vimentin. Briefly, tumor samples were fixed with 4% paraformaldehyde, embedded in paraffin, and sliced into 4 μm thin sections. After deparaffinization and rehydration, endogenous peroxidase activity was blocked with 3% H_2O for 20 min. Then the sections were incubated with rabbit polyclonal anti-α-SMA (Abcam, ab5694, 1:200) or

rabbit monoclonal anti-vimentin (Cell Signaling Technology, 5741S, 1:200) at 4°C overnight. After washing, the sections were then incubated with horseradish peroxidase-conjugated secondary antibodies at room temperature for 60 mins. Finally, the sections were counterstained with hematoxylin and visualized under a microscope (NIKON ECLIPSE TI, Japan).
